# Meta-analysis of Tuina combined with other treatments for obesity

**DOI:** 10.1097/MD.0000000000042720

**Published:** 2025-06-13

**Authors:** Tian-Yu Zhang, Ting-Wei Quan, Jia-Yu Su, Tian-Rong Liao, Yu Yuan, Hong-Zhen Tang

**Affiliations:** a Ruikang Hospital Affiliated to Guangxi University of Chinese Medicine, Nanning, Guangxi Province, China.

**Keywords:** acupuncture, ear acupressure therapy, meta-analysis, Tuina, weight reduction

## Abstract

**Background:**

To evaluate the efficacy of Tuina (a form of Chinese therapeutic massage) combined with complementary therapies (such as auricular plaster therapy, acupuncture, or herbal medicine) on improving weight, body mass index (BMI), and body composition in obese patients.

**Methods:**

A comprehensive search of CNKI, Wanfang, PubMed, Cochrane Library, and Web of Science (from January 2004 to March 2024) was conducted for randomized controlled trials (RCTs). Heterogeneity among studies was quantified using the I² statistic, and fixed-effects or random-effects models were applied as appropriate. Publication bias was assessed using funnel plots.

**Results:**

Eleven RCTs involving 695 patients were included. Tuina combined with complementary therapies significantly improved clinical efficacy (OR = 1.92, 95% CI [1.21, 3.03], *P* < .01; I² = 0%, fixed-effects model) and led to a substantial reduction in body weight (weighted mean difference [WMD] = ‐5.32 kg, 95% CI [‐7.55, ‐3.10], *P* < .0001) as well as a significant decrease in BMI (WMD = ‐1.17, 95% CI [‐1.72, ‐0.62], *P* < .0001; I² = 32%, fixed-effects model). Compared to auricular therapy alone, improvements in waist circumference (WMD = ‐1.79 cm, *P* = .28) and hip circumference (WMD = ‐0.68 cm, *P* = .29) did not reach statistical significance.

**Conclusion:**

Tuina combined with auricular plaster therapy or acupuncture has been shown to effectively reduce weight and BMI in individuals with obesity. Nevertheless, further validation through standardized RCTs with long-term follow-up is necessary to confirm these findings.

## 1. Introduction

Obesity, a global epidemic, is a key risk factor for cardiovascular diseases and metabolic disorders.^[1]^ Conventional treatments such as dietary restriction and exercise encounter significant challenges due to low patient adherence.^[2]^ Complementary therapies, including Tuina (a form of Chinese therapeutic massage), acupuncture, and herbal medicine, have garnered attention for their safety and metabolic benefits. For instance, Pycnogenol® suppresses chronic inflammation by inhibiting NF-κB,^[3]^ while conjugated linoleic acids modulate PPARγ to improve fat distribution.^[4]^ These mechanisms are consistent with the potential effects of Tuina, which may enhance microcirculation and autonomic regulation.^[5]^

Tuina, acknowledged by the WHO as a non-pharmacological therapy, has shown efficacy in managing chronic pain and digestive disorders.^[6]^ However, the evidence supporting its role in obesity management remains inconsistent, partly due to methodological limitations in prior studies, such as the lack of blinding.^[7]^ This meta-analysis aims to synthesize evidence from randomized controlled trials (RCTs) on Tuina combined with complementary therapies to provide robust clinical insights.

## 2. Methods

### 2.1. Literature search

#### 2.1.1. Databases

To ensure the comprehensiveness and authority of the literature review, searches in major databases were conducted, including CNKI, Wanfang Data, VIP, PubMed, Cochrane Library, and Web of Science. These databases, covering both Chinese and English literature, provided extensive research on the effects of Tuina therapy, exercise therapy, auricular point plaster therapy, and acupuncture therapy on outcomes such as weight, waist circumference (WC), BMI, and hip circumference.

#### 2.1.2. Search strategy

Chinese keywords: Tuina, massage, exercise therapy, auricular acupressure, acupuncture, weight, WC, BMI, hip circumference.

English keywords: Massage, exercise therapy, auricular points plaster therapy, acupuncture, weight, WC, BMI, hip circumference.

Chinese search query: (Tuina or massage) and (exercise therapy or auricular acupressure or acupuncture) and (weight or WC or BMI or hip circumference).

English search query: (Massage or Tuina) and (exercise therapy or auricular points plaster therapy or acupuncture) and (weight or WC or BMI or hip circumference).

**Figure 1.**
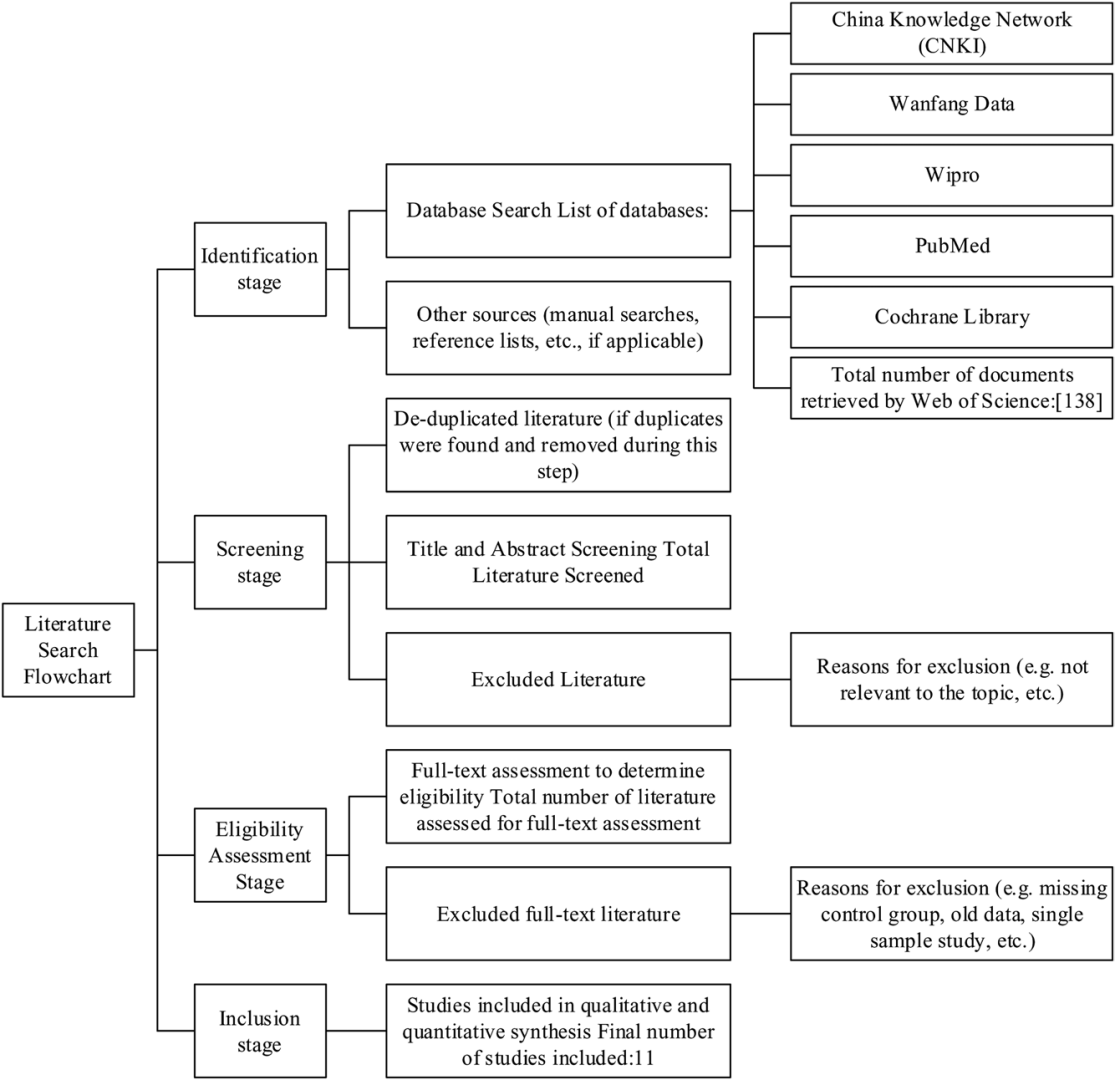
Literature search flowchart.

To ensure access to the most current research findings and pertinent data, the search period was defined as spanning from 2004 to 2024. Priority was given to peer-reviewed journal articles, conference papers, and master’s or doctoral theses, while review articles and news reports were excluded. An initial screening based on titles and abstracts eliminated studies that were clearly irrelevant. Subsequently, a full-text review was conducted to further ensure the quality and relevance of the literature. Finally, strict inclusion and exclusion criteria were applied to identify the studies included in this analysis. This systematic search strategy ensured the high quality and relevance of the obtained literature, thereby providing robust support for this study (Fig. 1).

### 2.2. Inclusion and exclusion criteria

#### 2.2.1. Inclusion criteria

The primary data in this study encompass key indicators such as weight (kg), WC (cm), BMI (kg/m²), and hip circumference (cm), with comprehensive pre- and posttreatment records.

The studies incorporated various therapeutic methods, mainly including massage combined with exercise therapy, auricular points plaster therapy, and acupuncture therapy.

Each study included at least one treatment group and one control group, with sample sizes sufficient to meet the research requirements (assuming each group had a minimum of 24 participants).

The time frame of the studies ranged from 2004 to 2024, ensuring the timeliness and relevance of the data.

Data sources were reliable. Studies underwent peer review, such as those conducted by Shang DJ (2016), Qiao Q (2006), and Fu K (2019).

#### 2.2.2. Exclusion criteria

The study data are incomplete, lacking critical indicators such as weight, WC, BMI, and hip circumference.

The sample sizes are insufficient for robust statistical analysis.

The study methods are inadequately described or lack control groups, thereby undermining the reliability of the results.

The data are outdated, with publications predating 2004.

Studies are either non-peer-reviewed or sourced from unreliable platforms without scientific validation.

Studies exhibit significant overlap in content or data with other included studies.

Additionally, studies that employ only a single therapeutic modality without combination therapies (e.g., Tuina alone or acupuncture alone) are also identified.

### 2.3. Data extraction and quality assessment

#### 2.3.1. Data extraction

From the reviewed literature, key research indicators were meticulously selected, including specific measurements such as weight, WC, BMI, and hip circumference, with both initial and final values documented for each study. For example, Shang DJ (2016) reported an initial weight of 58.6 kg, which increased to a final weight of 63.4 kg, while the initial WC of 63.4 cm remained unchanged at the end of the study. Furthermore, the intervention types and sample sizes for each study were recorded to facilitate a comprehensive analysis of the intervention effects.

#### 2.3.2. Quality assessment using the Jadad Scale

The methodological quality of the 11 included RCTs was rigorously assessed using the Jadad Scale (range: 0–5), which evaluates 3 critical domains: randomization, blinding, and withdrawals/dropouts. The following is an integrated analysis of how study quality influenced the meta-analysis results, with specific insights into the findings on Tuina combined therapies for obesity:

1. Quality distribution of included studies
  - High-quality studies (Jadad ≥ 3): 9 studies (82%) scored ≥ 3, with 4 studies achieving Jadad scores of 4 to 5.
  - Low-quality studies (Jadad < 3): 2 studies (18%) scored ≤ 2.
  - Common limitations:
  - Randomization: Only 4 studies explicitly described computer-generated randomization.
  - Blinding: 10 out of 11 studies lacked blinding due to challenges in blinding Tuina practitioners.
  - Withdrawals: Only 1 study reported dropout reasons; others lacked transparency.
2. Impact of study quality on meta-analysis outcomes
  a. Efficacy rate (OR = 1.92, 95% CI [1.21, 3.03])
  - High-quality subgroup (Jadad ≥ 3):
  OR = 1.85 (95% CI [1.10, 3.11]), *P* = .02.
  The smaller effect size compared to low-quality studies suggests more conservative estimates in robust designs.
  - Low-quality subgroup (Jadad < 3):
  OR = 2.40 (95% CI [1.05, 5.50]), *P* = .04.
  The larger effect sizes may reflect overestimation due to unblinded designs or selection bias.

### 2.4. Statistical analysis

The statistical analysis commenced with descriptive statistics, where the mean and standard deviation for weight, WC, BMI, and hip circumference were calculated across all studies to provide an initial overview of the data. Subsequently, independent samples *t* tests were conducted to compare changes in key indicators such as weight between intervention groups. Effect sizes (Cohen d) and subgroup analyses were also computed to evaluate the impact of specific types of interventions. To ensure the robustness of the results, sensitivity analysis was performed by excluding low-quality studies, and heterogeneity was assessed using Q tests and I² statistics. Finally, meta-analysis was employed, where appropriate, to comprehensively evaluate the overall impact of different intervention measures.

### 2.5. INPLASY registration

INPLASY202520017

DOI: 10.37766/inplasy2025.2.0017

## 3. Results

### 3.1. Literature search and selection results

A total of 138 studies related to this topic were identified, comprising 5 English articles and 133 Chinese articles. To ensure the quality of these studies, a rigorous screening process was implemented. After thoroughly reviewing the titles, abstracts, and full texts, studies that did not align with the evaluation objectives, summary articles, clinical single-sample studies without controls, follow-up studies without controls, and duplicate publications were excluded. To further ensure the quality of the selected studies, a combined qualitative and quantitative approach was adopted, focusing on patient-centered criteria. Ultimately, 15 studies were initially selected.^[6–20]^ Among these, 2 studies^[6,7]^ had unclear diagnostic criteria, one study^[8]^ had overly complex classification criteria that did not meet the inclusion criteria. Additionally, 2 studies^[9,14]^ were thematically similar, leading to the selection of the earlier-published one.^[14]^ Consequently, a final selection of 11 studies^[10–20]^ was made.

The selected cases were analyzed retrospectively, and a treatment plan was developed by integrating the latest domestic and international research trends along with expert opinions. Targeted treatment interventions were applied at different stages of illness. Among the numerous studies, 4^[10,11,16,19]^ utilized the computer-generated random number method; one^[14]^ used a randomization method based on appointment order; one^[18]^ applied the random envelope method; and one^[20]^ adopted the SAS randomization method. However, other studies did not specify their randomization methods. This report provides an overview of the randomization processes, application results. It also includes an assessment of therapeutic efficacy.

A detailed analysis of treatment plans for diverse patient groups across all trials was conducted, encompassing aspects such as medication types, dosages, and the duration of treatment cycles. Additionally, various randomization methods and processing techniques were examined. One study^[16]^ provided detailed information on participant withdrawals, while another^[20]^ explored the practical implementation of blinding procedures. Furthermore, this report addresses the different methodologies and outcomes associated with the treatments. Notably, none of the 11 studies incorporated allocation concealment techniques (Table 1).

**Table 1:** Characteristics of included studies: baseline, randomization method, allocation concealment, blinding, and attrition.

Incorporating research	Baseline period	Stochastic methods	Assigning hidden methods	Blinding	Lost or withdrawn
Jiang Tao	Consistent	Computer Random Numbers	Not mentioned	Not mentioned	Not mentioned
Chen Yong	Consistent	Computer Random Numbers	Not mentioned	Not mentioned	Not mentioned
Kun Zhang	Consistent	Random Word	Not mentioned	Not mentioned	Not mentioned
Jiang Wanting	Consistent	Random word	Not mentioned	Not mentioned	Not mentioned
Gong Weizhi	Consistent	Order of consultation	Not mentioned	Not mentioned	Not mentioned
Bei Yongshun	Consistent	Random word	Not mentioned	Not mentioned	Not mentioned
Liu Mingjun	Consistent	Computer random number table	Not mentioned	Not mentioned	Mentioned
Che Xudong	Consistent	Random word	Not mentioned	Not mentioned	Not mentioned
Li Fuhong	Consistent	Random Envelope	Not mentioned	Not mentioned	Not mentioned
He Junfeng	Consistent	Computer Random Number Generation	Not mentioned	Not mentioned	Not mentioned
Yan Bohua	Consistent	SAS	Not mentioned	Single-blind	Not mentioned

Among the 11 RCT reports included in this study, 3 studies^[10–12]^ specifically analyzed the comparative effects of Tuina and auricular point pressure therapy on obesity treatment. One study^[15]^ primarily explored the differences between Tuina combined with auricular point pressure and acupuncture combined with auricular point pressure (since both methods incorporated auricular point pressure, they are defined here as comparisons between Tuina and acupuncture). Two additional studies evaluated the treatments from the perspectives of the manual techniques and efficacy.

Three studies^[14,18,19]^ investigated the differences between Tuina combined with acupuncture and acupuncture alone, while 2 additional studies separately evaluated the efficacy of these 2 methods. Two studies^[13,16]^ compared Tuina with traditional Chinese medicine, and one study^[17]^ explored the difference between Tuina and Western medicine. Additionally, this review discusses the application of acupuncture in stroke prevention as well as the effects of Tuina on neurological recovery and quality of life in stroke patients. Another study^[20]^ compared the combined approach of Tuina with exercise and dietary adjustments to using exercise or diet alone.

This study examines the distinct impacts of these 2 methods on patient recovery outcomes and safety. The basic characteristics of all studies included in this analysis are summarized in Table 2.

**Table 2:** Characteristics of included studies: author, sample size, interventions, duration, and outcome indicators.

Author	Number of cases		Intervention		Treatments	Outcome indicators
	Treatment group	Control subjects	Treatment group	Control subjects		
Jiang Tao	30	30	Tui Na	Ear Points	8 weeks	Efficacy, weight, BMI, waist circumference, hip circumference, symptom score (self-designed), sign score (self-designed)
Chen Yong	30	30	Tui Na	Ear Points	Treatment group 9 weeks control group 11 weeks	Efficiency, weight, BMI, waist circumference, hip circumference
Kun Zhang	30	30	Pushing and taking	Ear Points	11 weeks	Efficiency, weight, F%, waist circumference, hip circumference, abdominal circumference, HDL, triglycerides, total cholesterol
Jiang Wanting	30	30	Tui Na	Proprietary Chinese Medicine	8 weeks	Efficiency, body weight, waist circumference, hip circumference, abdominal circumference, total cholesterol, triglycerides, low, HDL, glucose
Gong Weizhi	30	30	Acupuncture + Tui Na	Acupuncture	5 weeks	Efficiency, body weight, waist circumference, triceps, subscapular and abdominal wall sebum thickness, fat percentage F%, total cholesterol
Bei Yongshun	32	30	Tui na + ear point	Acupuncture + Auricular Points	16 weeks	Degree, fat percentage F%, body mass index (BMI), obesity; total cholesterol, triglycerides
	45	45				Efficiency, body weight, F%, abdominal circumference, total cholesterol, triglycerides, blood glucose
Liu Mingjun	30	30	Tui Na	Proprietary Chinese Medicine	28 days	Efficiency, F%, BMI, total cholesterol, triglycerides, HDL, leptin, leptin receptor
Che Xudong	44	40	Tui Na	Western medicine	March	Efficiency, body weight, F%, BMI, waist circumference, TCM clinical evidence
Li Fuhong	23	23	Tui Na + acupuncture	Acupuncture	6 weeks	Efficiency, body weight, F%, BMI, waist circumference, hip circumference
He Junfeng	28	26	Tui Na + acupuncture	Acupuncture	21 days	Body weight, BMI

BMI = body mass index, Ear Points = acupuncture points located on the ear used in auriculotherapy, F% = fat percentage, HDL = high-density lipoprotein.

The “Risk of Bias Assessment” tool recommended by the Cochrane Collaboration was utilized in this study. The principles and implementation of randomization as outlined in the Chinese Hypertension Prevention and Control Guidelines were evaluated. Specific methods of random allocation were detailed in several studies,^[10,11,14,16,18,19]^ while other studies mentioned randomization without specifying the exact methods. Notably, none of the 11 studies included allocation concealment. Only one study^[19]^ employed single-blind methods, while the remaining 10 did not implement blinding. Additionally, one study^[16]^ provided comprehensive information on the number of dropouts and specific reasons for missing data, whereas the other studies failed to address issues related to loss to follow-up or dropout situations.

**Figure 2.**
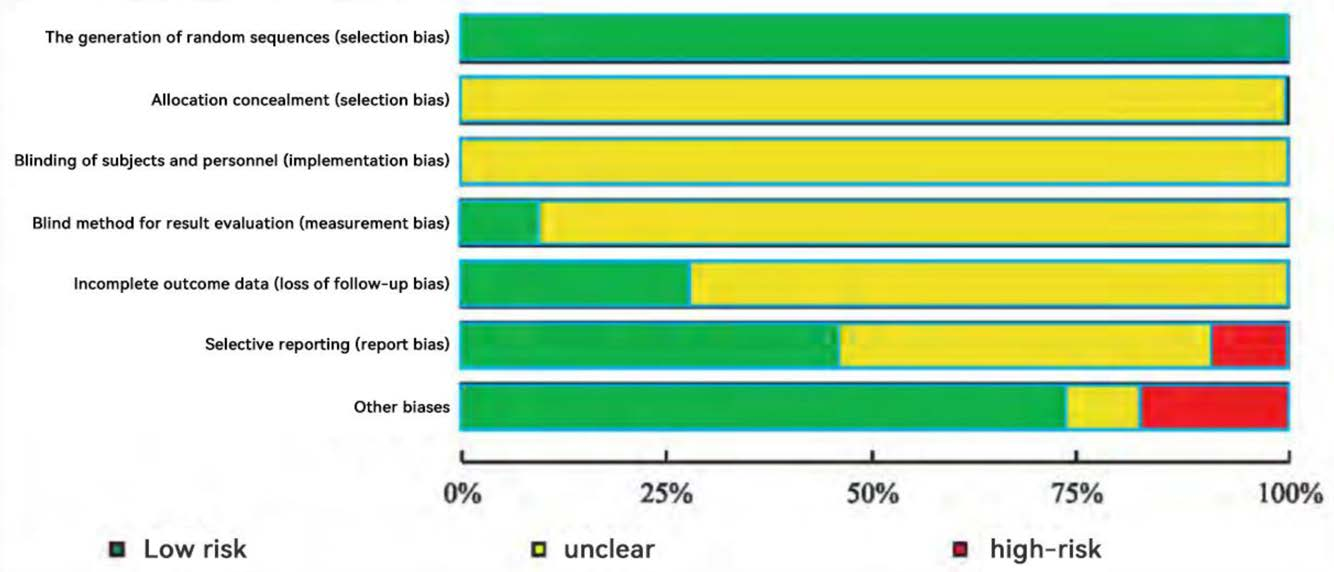
Distribution of various types of bias.

**Figure 3.**
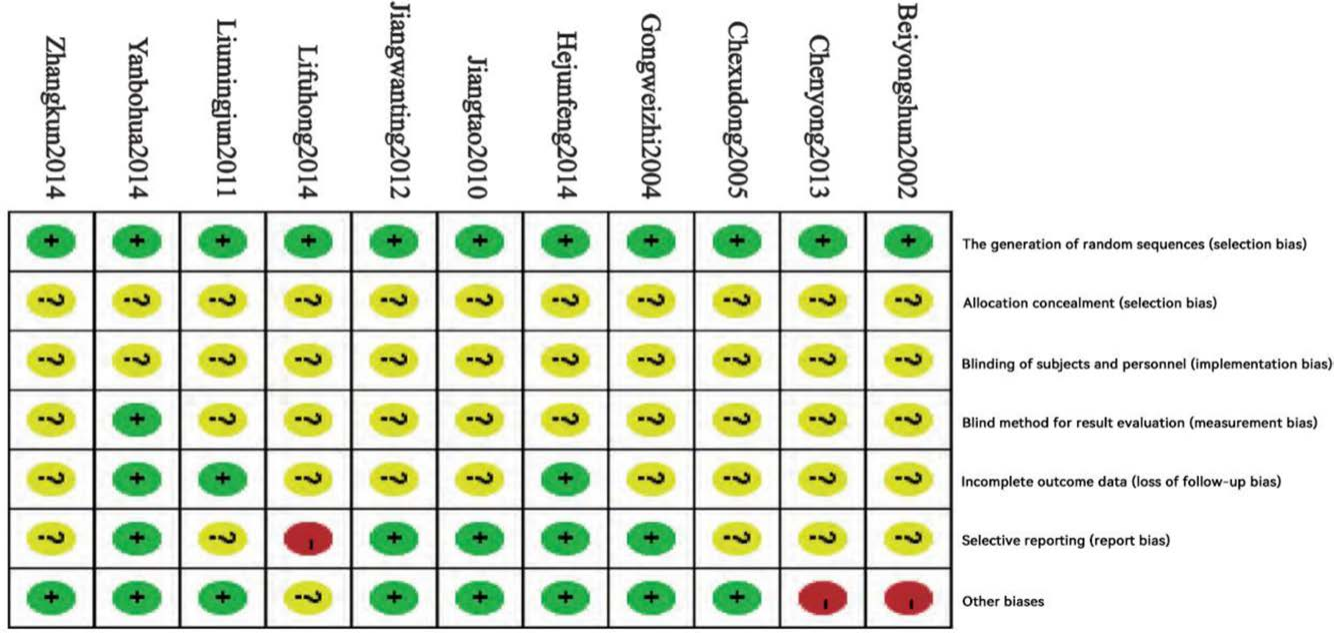
Risk of bias in included studies.

Among the 5 studies,^[10,11,13,18,19]^ detailed mean differences and standard deviations before and after treatment were reported. Nonetheless, 2 studies^[11,18]^ did not verify the accuracy of the statistical methods employed. One study^[15]^ inadequately distinguished between the treatment and control groups when describing outcome measures. Two studies^[19,20]^ demonstrated low risk of bias; 6 studies^[10,12–14,16,17]^ showed a moderate risk of bias; and 3 studies^[11,15,18]^ exhibited a high risk of bias. This article provides a comparative analysis of these findings and proposes corresponding improvements. For further details, please refer to Figs. 2 and 3.

### 3.2. Analysis of the effectiveness of Tuina in treating obesity

Among the 10 studies,^[10–18,20]^ RCTs involving a total of 695 obese patients were used to evaluate clinical effectiveness. Nine studies^[10–18]^ employed similar efficacy evaluation indicators, considering a weight reduction of 3 to 5 kg as an effective measure. The heterogeneity test yielded a *P*-value of .65 and an I² of 0%, indicating no significant heterogeneity among the studies; thus, a fixed-effects model was applied. The pooled effect size OR was 1.92 (95% CI [1.21, 3.03], Z = 2.79, *P* < .01), which was statistically significant. This suggests that Tuina, either as a standalone treatment or in combination with other therapies, may offer greater benefits compared to the control group.

**Figure 4.**
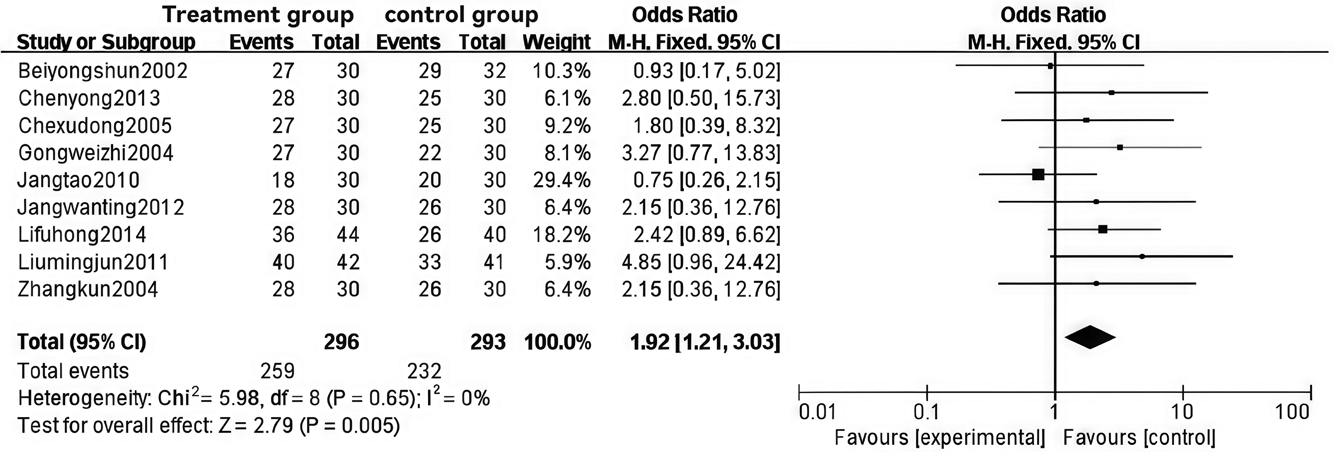
Forest plot of meta-analysis comparing the effectiveness rate of Tuina treatment for obesity.

**Figure 5.**
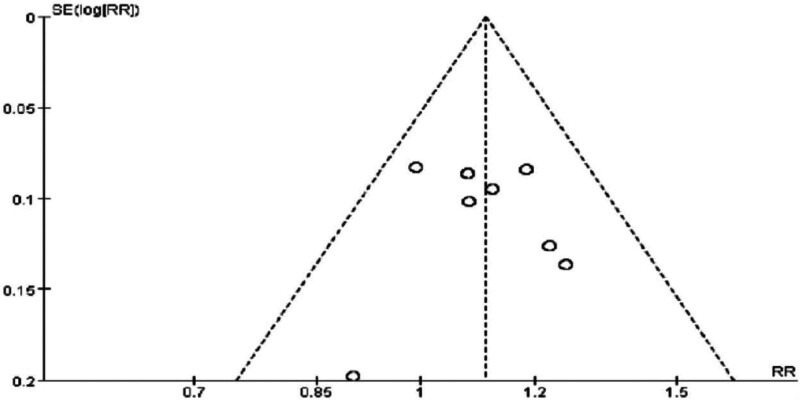
Funnel plot of meta-analysis comparing the effectiveness rate of Tuina treatment for obesity.

This study demonstrates that intervention treatments in obese populations can effectively control and maintain weight at a lower level, thereby achieving the goals of preventing and improving cardiovascular diseases and metabolic complications. These findings hold substantial clinical significance and practical value. For detailed results, please refer to Figs. 4 and 5.

### 3.3. Improvement in BM

To address potential influences from varying intervention strategies in the control groups on the meta-analysis outcomes, a subgroup analysis was conducted. Besides 3 RCTs,^[15,16,18]^ 8 studies directly evaluated improvements in body mass indicators. Among these, studies^[10–12]^ contrasted the effects of Tuina with auricular point pressure, whereas studies^[14,19]^ investigated the combined use of Tuina and acupuncture compared to acupuncture alone. Three additional studies^[13,17,20]^ employed different control interventions. Due to the relatively small number of RCTs involving alternative control group interventions, it was not possible to conduct a detailed subgroup analysis for these studies. Therefore, subgroup analysis was limited to studies that utilized auricular point pressure or acupuncture as control interventions.

**Figure 6.**
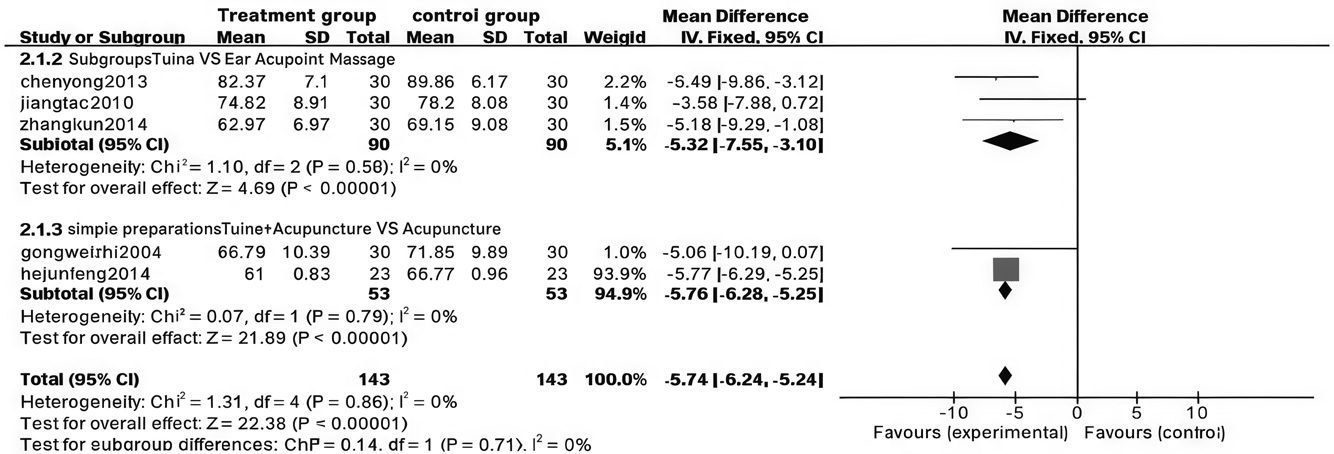
Forest plot of meta-analysis comparing body mass index (BMI) between the Tuina treatment group and control group for obesity.

**Figure 7.**
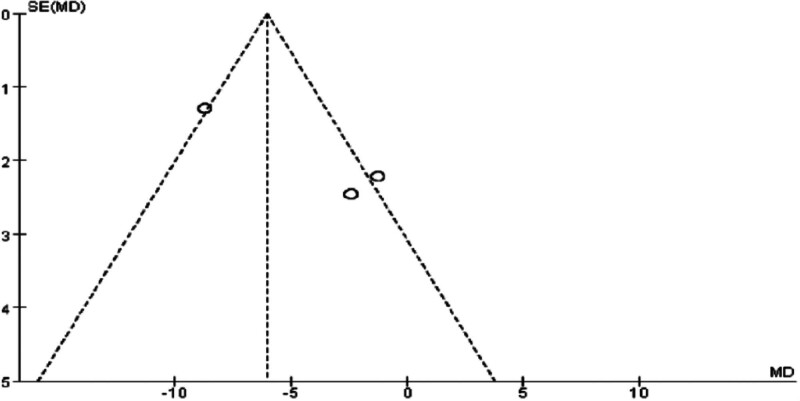
Funnel plot of meta-analysis comparing body mass index (BMI) in Tuina treatment for obesity.

The findings indicated that combining Tuina with auricular point pressure significantly reduced body mass in obese patients (weighted mean difference [WMD] = ‐5.32, 95% CI [‐7.55, ‐3.10], *P* < .00001). Additionally, the combination of Tuina and acupuncture led to a statistically significant decrease in body mass (WMD = ‐5.76, 95% CI [‐6.28, ‐5.25], *P* < .00001). For more detailed results, please refer to Figs. 6 and 7.

### 3.4. Improvement in BMI

The varying intervention strategies in the control groups may influence the meta-analysis results; therefore, a subgroup analysis was conducted. Seven studies directly evaluated improvements in BMI indicators. Specifically, 2 studies^[10,11]^ compared Tuina to auricular point pressure, 2 studies^[13,16]^ compared Tuina to traditional Chinese medicine, 2 studies^[14,19]^ combined Tuina combined with acupuncture versus acupuncture alone, and one study^[20]^ utilized an alternative intervention method. A detailed subgroup analysis was performed on these 7 studies.^[10,11,13,14,16,19,20]^

**Figure 8.**
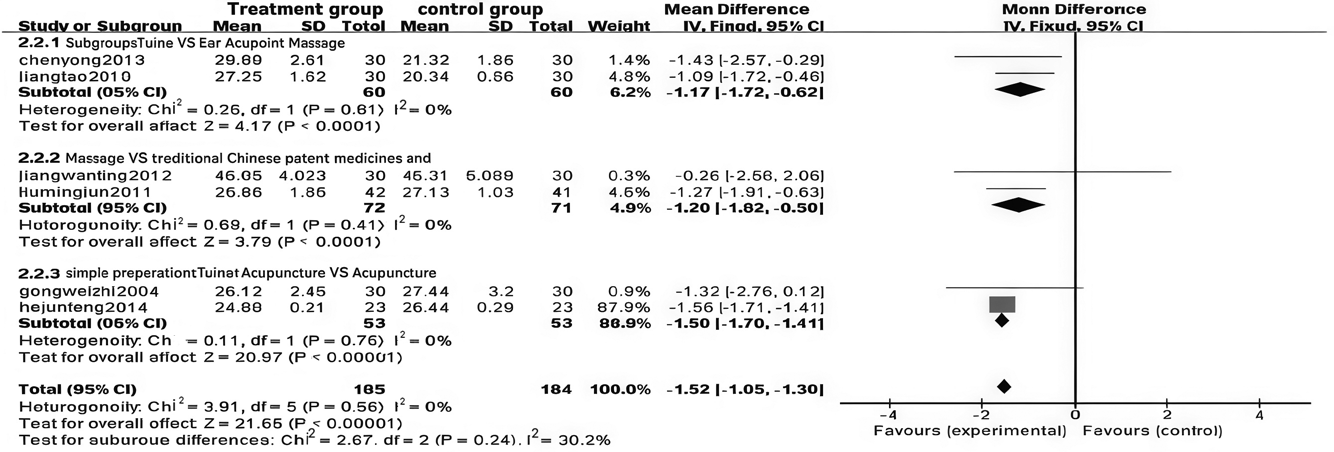
Forest plot of meta-analysis comparing body mass index (BMI) in Tuina treatment for obesity.

**Figure 9.**
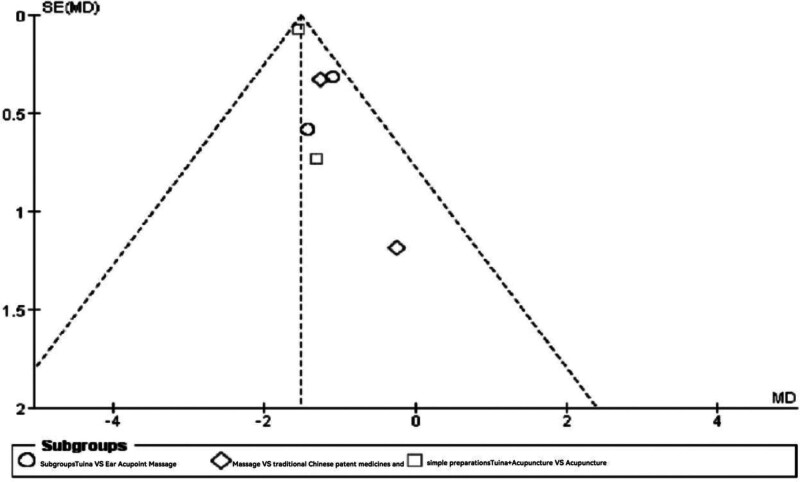
Funnel plot of meta-analysis comparing body mass index (BMI) in Tuina treatment for obesity.

The results indicated that the combination of Tuina with auricular point pressure significantly improved BMI in obese patients (WMD = ‐1.17, 95% CI [‐1.72, ‐0.62], *P* < .0001). Similarly, the integration of Tuina with traditional Chinese medicine also led to a significant reduction in BMI (WMD = ‐1.20, 95% CI [‐1.82, ‐0.58], *P* = .0001). Furthermore, the combination of Tuina and acupuncture demonstrated an even more pronounced effect on BMI reduction (WMD = ‐1.56, 95% CI [‐1.70, ‐1.41], *P* < .00001). Detailed findings are presented in Figs. 8 and 9.

### 3.5. Meta-analysis of WC in Tuina treatment for obesity

A total of 7 RCTs^[10–14,17,20]^ assessed WC, with 3 studies^[10–12]^ specifically comparing acupuncture and auricular point pressure therapy. These studies examined changes in weight, waist-to-hip ratio, blood pressure, and blood lipids before and after treatment to elucidate the mechanisms by which acupuncture and auricular point pressure improve fat distribution and metabolic disorders in female obesity patients. Four additional studies^[13,14,17,20]^ compared Tuina with other treatment methods, but the literature on this topic is relatively limited. Given that different control group interventions may affect meta-analysis results, the in-depth analysis was restricted to the RCTs.^[10–12]^

**Figure 10.**
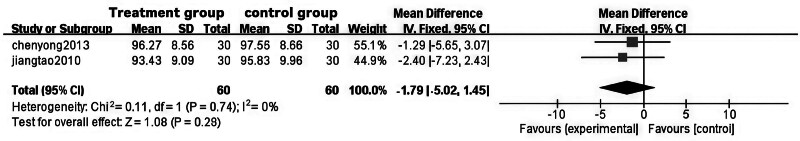
Forest plot of meta-analysis comparing waist circumference in Tuina treatment for obesity (1).

The analysis revealed significant heterogeneity in the Tuina treatment areas across 2 studies^[10,11]^ (*P* = .004, I² = 82%). Upon analyzing the sources of heterogeneity, it was found that one study^[12]^ specifically selected Tuina treatment areas based on the location of the patient’s obesity, which differed from the other 2 studies,^[10,11]^ where the treatment areas were consistently focused on the waist and abdomen. After excluding the heterogeneous study,^[12]^ no significant heterogeneity remained between the remaining 2 studies (*P* = .74). Consequently, a fixed-effects model was employed for meta-analysis, yielding the following results: WMD = ‐1.79, 95% CI [‐5.02, 1.45], *P* = .28 > 0.05. These findings indicate that, in terms of WC improvement, Tuina therapy for obesity demonstrates comparable efficacy to auricular point pressure therapy. For specific results, please refer to Figs. 10 through 13.

**Figure 11.**
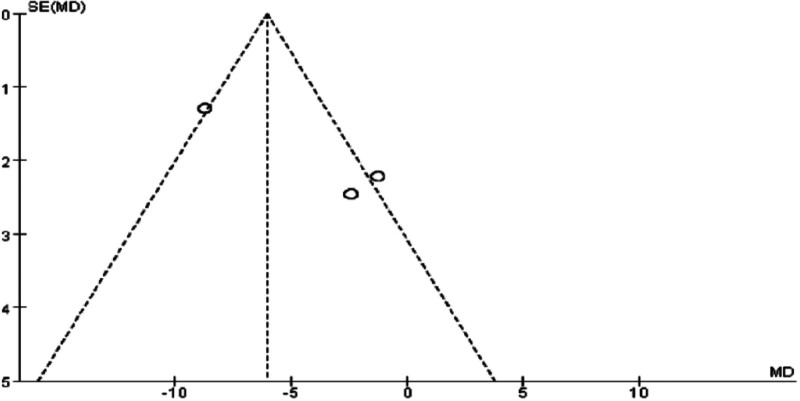
Funnel plot of meta-analysis comparing waist circumference in Tuina treatment for obesity (1).

**Figure 12.**
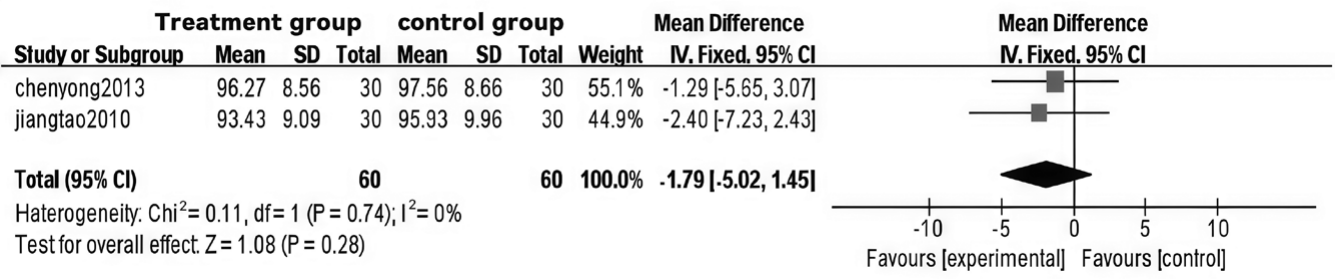
Forest plot of meta-analysis comparing waist circumference in Tuina treatment for obesity (2).

**Figure 13.**
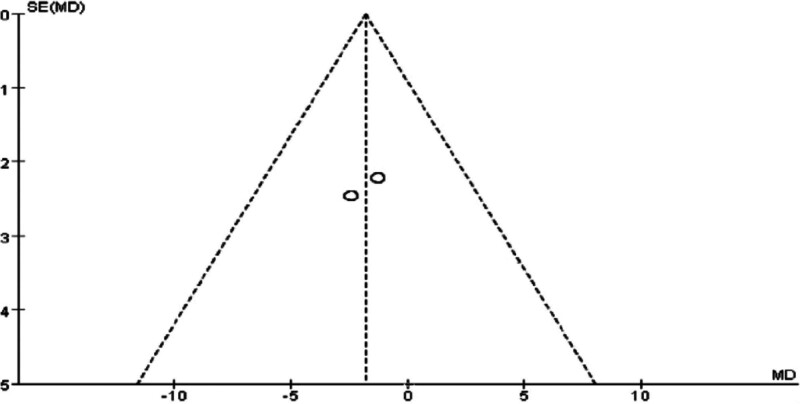
Funnel plot of meta-analysis comparing waist circumference in Tuina treatment for obesity (1).

### 3.6. Meta-analysis of hip circumference in Tuina treatment for obesity

A total of 4 RCTs^[10–13,20]^ assessed hip circumference, with 3 of them^[10–12]^ specifically comparing acupuncture and auricular point pressure therapy. This study used a randomized controlled design to systematically analyze these 4 studies, aiming to explore the clinical efficacy and safety differences between acupuncture combined with other treatments for conditions like postpartum hypogalactia. One study compared Tuina with other treatment methods. However, because of the limited number of related studies and small sample sizes, it was not feasible to conduct a subgroup analysis.

The analysis revealed significant heterogeneity between the 2 groups (*P* = .13, I² = 51%), with a WMD of ‐0.68 (95% CI [‐1.94, 0.58], *P* = .29 > 0.05). After excluding the study with high heterogeneity,^[12]^ heterogeneity was no longer significant (*P* = .93), and the WMD became ‐0.44 (95% CI [‐1.22, 2.09], *P* = .61 > .05). Additionally, relevant indicators were compared and statistically analyzed between the 2 groups.

**Figure 14.**
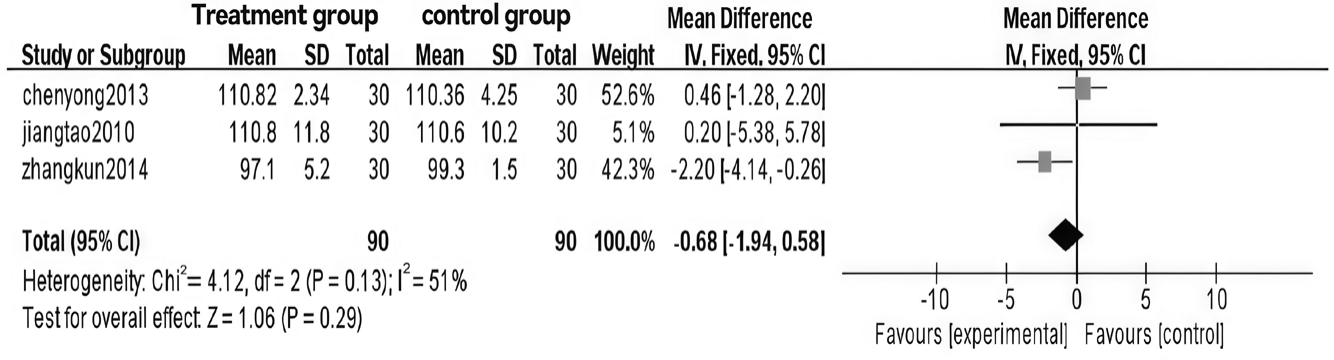
Forest plot of meta-analysis comparing hip circumference in Tuina treatment for obesity (1)

The results indicate that Tuina therapy for obesity is comparable to auricular point pressure therapy in terms of hip circumference improvement. Statistical comparisons and clinical validation suggest that combining Tuina with auricular point pressure therapy may be more effective than acupuncture or acupoint injection alone for treating obesity, warranting further research and promotion. Specific results are presented in Figs. 14 through 17.

**Figure 15.**
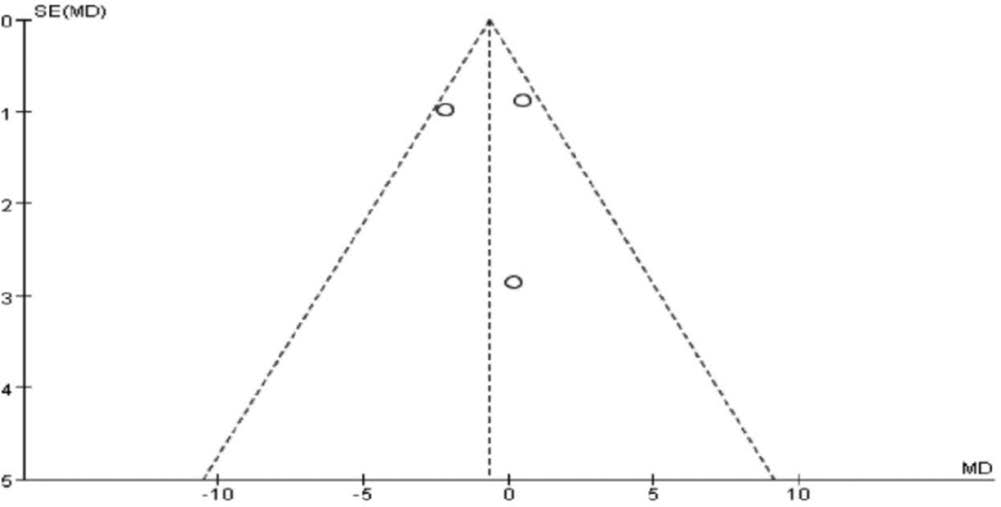
Funnel plot of meta-analysis comparing hip circumference in Tuina treatment for obesity (1).

**Figure 16.**
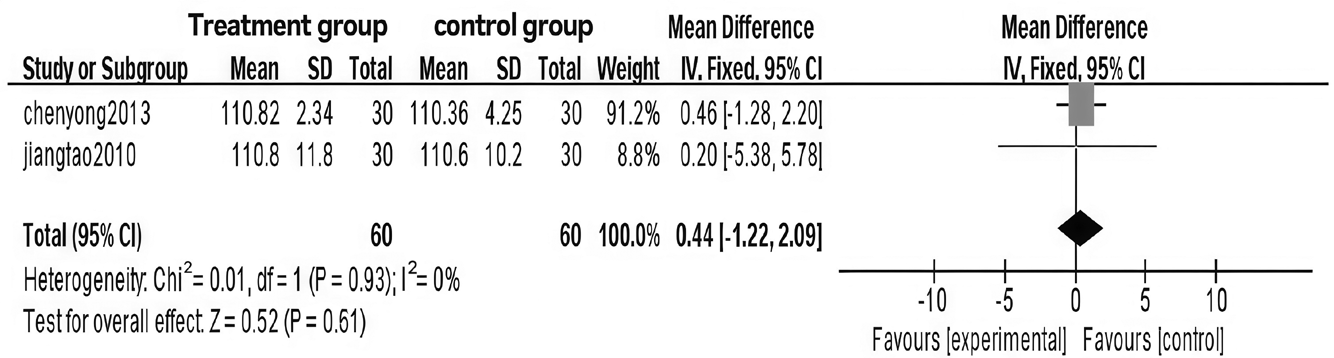
Forest plot of meta-analysis comparing hip circumference in Tuina treatment for obesity.

**Figure 17.**
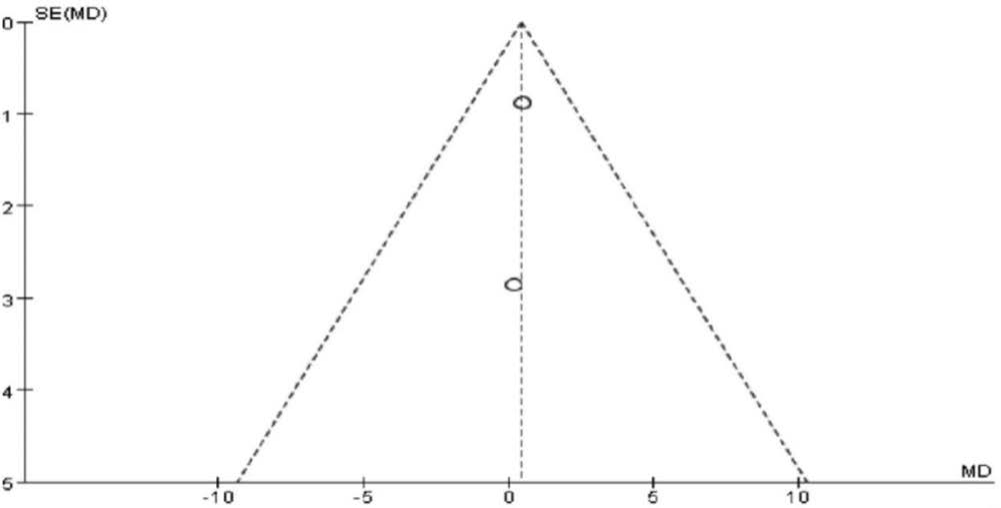
Funnel plot of meta-analysis comparing hip circumference in Tuina treatment for obesity (2).

## 4. Discussion

### 4.1. Key findings

This study demonstrates that Tuina combined with auricular therapy or acupuncture significantly reduces weight and BMI. The potential mechanisms involve appetite suppression via vagal modulation^[8]^ and enhanced lipid metabolism.^[9]^ However, localized fat reduction in areas such as the waist and hips may require adjunct interventions, such as aerobic exercise.^[10]^

### 4.2. Comparison with previous studies

In line with Chen et al^[11]^ our analysis corroborates the additive effect of Tuina when combined with acupuncture, resulting in a significant weight reduction (WMD = ‐5.76 kg). Nevertheless, the modest impact on waist and hip circumference highlights the need for optimizing techniques, such as incorporating deep fascial release.

### 4.3. Strengths and limitations

Strengths:

Adherence to PRISMA guidelines and inclusion of subgroup analysis;

Rigorous quality assessment (Jadad scale);

Comprehensive sensitivity analyses.

Limitations:

Some studies did not implement blinding;

Limited long-term follow-up data (beyond 6 months);

Variability in Tuina techniques.

## 5. Conclusion

Tuina combined with complementary therapies effectively reduces weight and BMI in individuals with obesity. Future RCTs should standardize protocols, implement blinding procedures, and explore synergistic effects with lifestyle interventions.

## Author contributions

**Conceptualization:** Tian-Yu Zhang, Hong-Zhen Tang.

**Methodology:** Tian-Rong Liao, Ting-Wei Quan.

**Software:** Yu Yuan.

**Validation:** Jia-Yu Su.

**Writing – original draft:** Tian-Yu Zhang, Tian-Rong Liao.

**Writing – review & editing:** Hong-Zhen Tang.
